# Probiotic viability – does it matter?

**DOI:** 10.3402/mehd.v23i0.18567

**Published:** 2012-06-18

**Authors:** Sampo J. Lahtinen

**Affiliations:** DuPont Nutrition and Health, Active Nutrition, Kantvik, Finland

**Keywords:** probiotics, non-viable probiotics, viability, efficacy, mechanisms

## Abstract

Probiotics are viable by definition, and viability of probiotics is often considered to be a prerequisite for the health benefits. Indeed, the overwhelming majority of clinical studies in the field have been performed with viable probiotics. However, it has also been speculated that some of the mechanisms behind the probiotic health effects may not be dependent on the viability of the cells and, therefore, is also possible that also non-viable probiotics could have some health benefits. The efficacy of non-viable probiotics has been assessed in a limited number of studies, with varying success. While it is clear that viable probiotics are more effective than non-viable probiotics and that, in many cases, viability is indeed a prerequisite for the health benefit, there are also some cases where it appears that non-viable probiotics could also have beneficial effects on human health.

Viability is an inherent property of probiotics since the current definition of probiotics, issued by the Joint FAO/WHO Working Group (1), defines that probiotics are ‘live microorganisms which, when administered in adequate amounts, confer a health benefit on the host’. Therefore, by definition, viability is an essential requirement for probiotics. This does not necessarily implicate that viability is an essential requirement for the health benefits conferred by probiotics or their derivates. As indicated later, there may be situations in which the health benefits of probiotics do not necessarily depend on the viability status of the cells – despite that it is widely acknowledged that, in general, viable probiotics are more effective than non-viable probiotics and that the health effects of viable probiotics have been explored to far greater extent than the potential health effects of non-viable probiotics. Research (and reviews) on this topic are hampered by lack of satisfactory terminology. No proper term or definition exists for the non-viable forms of probiotics. Terms such as non-viable probiotics and inactivated probiotics have been used, but these terms are self-contradictory since the word ‘probiotic’ as such indicates viability. In this discussion, the term non-viable probiotics is used in the lack of better terminology.

## Mechanisms of probiotic health effects – is viability essential?

While probiotics have been linked with different health benefits in a plethora of clinical trials with a variety of different outcomes, study populations, and probiotic ingredients, it is acknowledged that, in most cases, the exact mechanisms of the health benefits are not fully understood. Mechanistic studies have provided several plausible and possible modes of action, but in many cases, it has not been possible to identify direct and undisputed cause-effect relationships. In many cases, there are several potential mechanisms that could explain a certain clinical health benefit, and it has not been easy to exclude the other potential mechanisms in favour of a single mode of action. Perhaps, this is only natural, since a clinical health benefit may be a combined result of a number of different mechanistic effects occurring at cellular and molecular levels. The potential health efficacy of non-viable probiotics depends on whether the mechanism of the probiotic health effect itself is dependent on the viability of the cells. Given that there are multiple potential mechanisms, it is clear that this consideration should be taken case-by-case.

Adhesion to host tissues is thought to facilitate the host–microbial interactions such as the effects of microbes on the immune system of the host. Therefore, adhesion may be a key determinant for probiotic efficacy. In the gut, administered probiotics are clearly outnumbered by the resident gut microbiota. As such, this may reduce the chances of probiotics for having a major effect on the host health – however, adhesion to host mucosa may change the balance in the favour of probiotics locally and temporarily. Thus, at mucosal level, probiotics may become a major member of the local microbial population and become an important effector of host–microbial interactions. This may be particularly relevant in the small intestine, where the resident microbial numbers are smaller than in the colon. The effect of viability on adhesion is not fully understood and may be strain dependent (2). Some reports suggest that viable and non-viable lactobacilli are equally adherent to intestinal mucus (3). The adherence may be dependent on the way by which the cells have been killed; one study suggested that heat-killing and protease treatments were detrimental to the ability of probiotics to adhere to human mucus, but other means of cell killing had no effect (4). An *in vivo* mouse study suggested that heat-killing of lactobacilli affects the localization of the cells in the intestine; viable bacteria were reported to be located in the Peyer's patches and lamina propria shortly after administration to mice, whereas most heat-killed bacteria were located in the lumen and were rapidly cleared (5). While adhesion to host tissues may be equally efficient between viable and non-viable bacteria, prolonged colonization in the mucosa obviously requires formation of a viable colony. Production of antimicrobial compounds is one potential mechanism of probiotic action against pathogens and clearly a property of viable bacteria only. However, in addition to *in situ* production in the intestine, antibacterial compounds may also be produced during manufacturing process and then used as bacterial lysates or extracted ingredients. It has also been suggested that heat-killed lactobacilli may inhibit pathogen adhesion to host tissues by competitive exclusion (6).

Reduction of gut permeability is another potential mechanism of probiotic action, which has been reported for several viable probiotics, although mainly in cell cultures or in animal models. The molecular mechanisms by which the integrity of the epithelial layer is improved are not fully understood. It is known that production of short chain fatty acids such as acetic acid improves the epithelial integrity locally. Clearly, *in situ* production of short chain fatty acids is a property of a viable cell only. While research assessing the efficacy of non-viable probiotics is minimal, some studies have suggested that inactivated lactobacilli (7) and cell-free supernatants of probiotics (8) may improve epithelial integrity.

Interactions between probiotics and host immune system have been investigated in numerous studies with viable probiotics, but in many cell culture studies, non-viable probiotics have also been used. Probiotic cell components associated with *in vitro* immunomodulatory properties include cell wall extracts (9), lipoteichoic acids (10), bacterial DNA (11, 12), and S-layer proteins (13). Some clinical studies have also suggested that non-viable probiotics can modulate human immune system, e.g. by enhancing salivary IgA production (14) and by modulating host T-cell responses (15) and gene expression (16). Limited number of *in vitro* and animal studies have directly compared the effects of viable and inactivated probiotics on innate immunity, and in many cases, these have been found to be equally effective (17–19). A study by Gill and Rutherfurd (20) suggested that viable and killed cells of *Bifidobacterium lactis* HN019 were able to enhance cell phagocytic responses in mice peripheral blood cells, but only viable cells increased the phagocytic activity of peritoneal cells. In some studies, viable probiotics have proved to be more effective than non-viable probiotics (21–23). In the case of adaptive immunity, most studies comparing the two have favoured viable probiotics (5, 20, 24–26). However, one study suggested that both viable and killed *Lactobacillus* cells are able modulate the phenotype and functions of human myeloid dendritic cells (27).

In conclusion, many potential mechanisms of probiotic action are clearly dependent on cell viability and activity, but there is preclinical evidence suggesting that some mechanisms associated with probiotics may not be directly dependent on cell viability. These include adhesion to host tissues and modulation of innate immune responses. However, *in vivo* situation may be different and viability may be an indirect determinant of the health effect, since viable probiotics may be more likely to reach the site of action in the first place and remain at the site long enough to confer a health benefit.

## Clinical benefits of probiotics – is viability essential?

Probiotic microbes have been linked with a range of beneficial effects on host health. By far, most of the health efficacy documentation has been generated using viable probiotics, and there are too few data to make firm conclusions on the clinical efficacy of non-viable probiotics. Nevertheless, some studies have been carried out using different non-viable probiotics.

Gut health is the most important target for probiotics. Prevention and treatment of different forms of diarrhoea is one of the most successful and best documented health benefits of viable probiotics, but efficacy studies with non-viable probiotics are rare. One study suggested that a treatment with heat-killed *Lactobacillus acidophilus* LB was effective, even more so than a treatment with viable non-specified strain of *L. acidophilus* (28). One study compared viable or heat-killed *Lactobacillus rhamnosus* GG and found no difference in their effect on diarrhoea duration, but the study lacked a proper placebo group (29). Ouwehand and Salminen (30) have earlier concluded that both viable and non-viable probiotics may be useful for short-term treatment or prophylactic treatment of diarrhoea, but viable probiotics are necessary for an enhanced immunological response. Irritable bowel syndrome is a popular target for probiotic research, but to date, the research has focused almost exclusively on viable probiotics. However, in one clinical study, heat-inactivated cells were used as controls for viable cells (31). The administration of the viable product resulted in subjective improvement of the symptoms in 80% of the patients, compared to 40% in the control group, suggesting that viable probiotics may be more efficient in the treatment of irritable bowel syndrome. While no clinical data suggest that probiotics alone would be efficient in eradicating *Helicobacter pylori*, both viable and non-viable probiotics have been reported to increase the eradication rates of a standard anti-*H. pylori* regimen (32, 33). Some studies have concluded that both viable and non-viable probiotics are equally effective in the treatment of *H. pylori* infections (34), which others have highlighted the importance of viability (35, 36).

Improvement of lactose digestion by probiotics deserves special attention in the context of viability. Most studies comparing the efficacy of live and dead lactobacilli in improving lactose digestion have been performed using yoghurt starter cultures, not probiotics. Most of the clinical studies comparing live and pasteurized yoghurt suggest that viable cells are more effective in improving lactose digestion (37, 38). However, cell viability as such may not be the critical factor for the efficacy. In one study, it was concluded that, to improve lactose digestion, the bacteria need not to be alive, but intact cell walls are required to protect the active *β*-galactosidase during gastrointestinal passage; the efficacy of pasteurized bacteria was low, but the effect of bacteria killed with gamma irradiation was similar to the effect of viable bacteria (39).

Prevention and treatment of allergic disease has been a popular target of probiotic research, and some studies have also included non-viable probiotics. In one small trial comparing viable and heat-inactivated *L. rhamnosus* GG in the management of infant atopic eczema and cow's milk allergy, the latter were associated with increased gastrointestinal symptoms (40). Moreover, one study reported fewer subjective allergy symptoms in adults consuming yoghurt containing viable bacteria compared to subjects consuming heat-inactivated yoghurt (41). On the other hand, certain reports have suggested that both viable and non-viable probiotics may be useful in the treatment of allergic rhinitis (42, 43). It is possible that probiotic viability is more important in the management of eczema compared to the management of allergic rhinitis.

Efficacy of probiotics in prevention and supportive treatment of cancer is challenging and far from elucidated. Nevertheless, some early reports have suggested that heat-killed *Lactobacillus casei* Shirota could be useful in the treatment of carcinoma of the uterine cervix (44, 45) and secondary to lung cancer (46). In one preclinical study, viable *L. casei* was found to be more effective than heat-killed *L. casei* in the prevention of secondary tumours in preimmunized mice (47). On the other hand, heat-killed lactic acid bacteria are more effective than viable bacteria in the binding of aflatoxin, a potent dietary carcinogen (48).

## Conclusions

Viability is an inherent property of probiotics, since the current definition of probiotics includes a requirement of viability. The definition of probiotics also includes a requirement of a health benefit. Probiotic viability has traditionally been thought to be a prerequisite for a health benefit. Indeed, in most cases, viable probiotics have proven to be more effective than inactivated probiotic products. Most importantly, the overwhelming majority of the clinical health efficacy research has been carried out with viable probiotics. Nevertheless, depending on the mechanism of action, there may be situations in which the health effects of probiotics are not dependent on the viability status of the cells, and there are some clinical reports suggesting efficacy of products containing inactivated probiotics. The research focussing on the importance of viability of probiotics is further complicated because – in a manner similar to other microbes – the viability of probiotics is not a simple on/off situation. For example, during storage in fermented probiotic products, part of the microbial population may become ‘dormant’, while other parts of the population may be already dead or still fully active and viable (49). The relevancy of these different subpopulations on the health efficacy of probiotics is unknown. There may also be a need to redefine the concept of viable in this context as several of the gut bacteria are viable but not culturable.
